# Impaired Clearance of Influenza A Virus in Obese, Leptin Receptor Deficient Mice Is Independent of Leptin Signaling in the Lung Epithelium and Macrophages

**DOI:** 10.1371/journal.pone.0108138

**Published:** 2014-09-18

**Authors:** Kathryn A. Radigan, Luisa Morales-Nebreda, Saul Soberanes, Trevor Nicholson, Recep Nigdelioglu, Takugo Cho, Monica Chi, Robert B. Hamanaka, Alexander V. Misharin, Harris Perlman, G. R. Scott Budinger, Gökhan M. Mutlu

**Affiliations:** 1 Department of Medicine, Division of Pulmonary and Critical Care Medicine, Northwestern University Feinberg School of Medicine, Chicago, Illinois, United States of America; 2 Department of Medicine, Division of Rheumatology, Northwestern University Feinberg School of Medicine, Chicago, Illinois, United States of America; University of California Los Angeles, United States of America

## Abstract

**Rationale:**

During the recent H1N1 outbreak, obese patients had worsened lung injury and increased mortality. We used a murine model of influenza A pneumonia to test the hypothesis that leptin receptor deficiency might explain the enhanced mortality in obese patients.

**Methods:**

We infected wild-type, obese mice globally deficient in the leptin receptor (*db/db*) and non-obese mice with tissue specific deletion of the leptin receptor in the lung epithelium (*SPC-Cre/LepR^fl/fl^*) or macrophages and alveolar type II cells (*LysM-Cre/Lepr^fl/fl^*) with influenza A virus (A/WSN/33 [H1N1]) (500 and 1500 pfu/mouse) and measured mortality, viral clearance and several markers of lung injury severity.

**Results:**

The clearance of influenza A virus from the lungs of mice was impaired in obese mice globally deficient in the leptin receptor (*db/db*) compared to normal weight wild-type mice. In contrast, non-obese, *SP-C-Cre^+/+^/LepR^fl/fl^* and *LysM-Cre^+/+^/LepR^fl/fl^* had improved viral clearance after influenza A infection. In obese mice, mortality was increased compared with wild-type mice, while the *SP-C-Cre^+/+^/LepR^fl/fl^* and *LysM-Cre^+/+^/LepR^fl^* ^/fl^ mice exhibited improved survival.

**Conclusions:**

Global loss of the leptin receptor results in reduced viral clearance and worse outcomes following influenza A infection. These findings are not the result of the loss of leptin signaling in lung epithelial cells or macrophages. Our results suggest that factors associated with obesity or with leptin signaling in non-myeloid populations such as natural killer and T cells may be associated with worsened outcomes following influenza A infection.

## Introduction

Obesity is a global health problem. In the US, 33% of adults are overweight (body mass index (BMI)>25 kg/m^2^) and 35% are obese (BMI>30 kg/m^2^) with a combined prevalence of 68% for overweight and obesity [1], [2]. It is estimated there are more than 1 billion adults who are overweight and at least 300 million people with obesity worldwide [3]. Although obesity is associated with increased morbidity and mortality due to increased risk of cardiovascular disease, diabetes, and asthma, it is also associated with a paradoxical protection against development of the acute respiratory distress syndrome (ARDS), which affects approximately 200,000 individuals and is associated with 75,000 deaths in the United States per year [4]. Similarly, in patients who develop ARDS, mortality is not increased and may be lower with increasing BMI [5]–[7]. In contrast, obesity was identified as an independent risk factor for increased hospitalization and death following influenza A infection during the 2009 H1N1 pandemic [8]–[10]. The mechanisms by which obesity might contribute to increased influenza A-induced ARDS and mortality are unknown [11]–[14].

Leptin is a 16-kDa protein encoded by the obese gene, whose primary function is to activate neurons in the hypothalamus that reduce food intake and energy expenditure [15]. Acquired leptin resistance, in which leptin levels are chronically elevated and the administration of exogenous leptin is less effective at inducing satiety, is a well-recognized feature of the metabolic syndrome [16]–[18]. Leptin also functions as a class I cytokine capable of activating the Janus kinase (JAK) and Signal Transducer and Activator of Transcription 3 (STAT3) signaling cascades [16]. We reasoned that impaired cytokine signaling through the leptin receptor might contribute to worsened lung injury in response to influenza A viral infection during obesity by reducing the rates of influenza A viral clearance. To test this hypothesis, we measured viral clearance and lung injury severity in mice globally deficient in leptin receptor signaling (*db/db*), which exhibit many features of the metabolic syndrome including obesity, hyperglycemia, and insulin resistance [19]. We compared these mice with normal weight mice in which the leptin receptor was deleted from the lung epithelium or macrophages.

## Materials and Methods

### Ethics statement

The protocol for the use of mice was approved by the Animal Care and Use Committee at Northwestern University (Protocol # 2009-1938 and 2011-1585). All procedures were performed under anesthesia, and all efforts were made to minimize suffering.

### Virus and cells

Influenza A virus (A/WSN/33 [H1N1]) was provided by Robert Lamb, Ph.D., Sc.D., Northwestern University, Evanston, IL. The A/WSN/33 (H1N1) virus is a mouse-adapted virus similar to A/Puerto Rico/8/1934 (H1N1) virus and is associated with significant morbidity (weight loss) and a dose dependent mortality in mice. Madin-Darby Canine Kidney (MDCK) cells (American Type Culture Collection (ATCC), Manassas, VA) cells were maintained in Dulbecco's modified Eagle's medium (DMEM) supplemented with 1% penicillin G/streptomycin and 10% fetal bovine serum (37°C, 5% CO_2_).

### Strains of mice

We used C57Bl/6 (wild-type mice at 8–12 weeks of age, 20–25 g) and *db/db* mice (mice with global leptin receptor deficiency at 8–12 weeks of age, 30–35 g) from Jackson Laboratories. We crossed mice with loxP sites in the leptin gene (*LepR^fl^* ^/fl^, a kind gift of Dr. Jeffery Friedman, Rockefeller University) with mice expressing Cre recombinase driven by the LysM promoter [20], [21] to generate *LysM-Cre^+/+^/LepR^fl^* ^/fl^ mice (mice with leptin receptor deficiency within macrophages and alveolar type II cells). We also crossed *LepR^fl^* ^/fl^ mice with transgenic mice expressing Cre recombinase driven non-conditionally by the surfactant protein C (SP-C) promoter (a kind gift from Dr. Brigid Hogan, Duke University) to generate *SP-C-Cre^+/+^/LepR^fl/fl^* mice [22]. The SPC promoter is expressed early during the development of the lung epithelium resulting in Cre mediated recombination in the entire airway and alveolar epithelium.

### Administration of influenza A virus

We anesthetized the wild-type C57Bl/6, *db/db*, *SP-C-Cre^+/+^/LepR^fl/fl^*, and *LysM-Cre^+/+^/LepR^fl^* ^/fl^ mice with isoflurane and intubated them using a 20-gauge angiocath cut to a length that placed the tip of the catheter above the carina [23], [24]. We then instilled either a mouse-adapted influenza A virus (A/WSN/33 [H1N1]) (1500 or 500 pfu/mouse, in 50 µL of PBS) or PBS (50 µL) as control as we have previously described [25].

### Clinical assessment of influenza A infection

We continuously observed mice infected with influenza A virus (A/WSN/33 [H1N1]) for signs of distress (slowed respiration, failure to respond to cage tapping, failure of grooming and fur ruffling). Mice that developed these symptoms were sacrificed and the death was recorded as an influenza A-induced mortality. Mice that died without developing these signs were also recorded as a mortality. Weight was measured on a daily basis.

### Collection of bronchoalveolar lavage (BAL) fluid for measurement of cell count and differential, protein, cytokines, and flow cytometry

We sutured a 20-gauge angiocath into the trachea via a tracheostomy and slowly infused a 1.0 mL aliquot of PBS into the lungs, which was aspirated three times. The aliquot was then immediately placed on ice. We then centrifuged the BAL fluid in a cytospin (2000 rpm for 10 minutes). We measured cell count and differential using a hemavet (Drew Scientific, Inc, Dallas, TX). We also measured BAL protein (Bradford), cytokines (BD Cytometric Bead Array Mouse Inflammation Kit, San Diego, CA), and interferon-α (VeriKine™ Mouse Interferon Alpha ELISA Kit, Piscataway, NJ) [26].

Flow cytometry measurements were performed as previously described [27]. Mice were euthanized with Euthasol followed by a rapid thoracotomy. The right ventricle was cannulated and while the right atrium was clamped with forceps, 5 ml of PBS was infused. The lungs were removed *en bloc* and the large airways were dissected from the peripheral lung tissue. The lung was minced and homogenized using Lung Dissociation kit (Miltenyl Biotek, Auburn, CA, Catalogue #130-095-927) according to the manufacturer's instructions for 30 minutes at 37°C and passed through 70 µm nylon mesh to obtain a single cell suspension. Remaining red blood cells were lysed using BD Pharm Lyse (BD Biosciences, San Jose, CA). Cells were counted using Countess automated cell counter (Invitrogen, Carlsbad, CA); dead cells were excluded using trypan blue.

After Live/Dead staining with Aqua dye (Invitrogen, Carlsbad, CA), cells were incubated with FcBlock (BD Biosciences, San Jose, CA) and stained with mixture of fluorochrome conjugated antibodies. Data were acquired on BD LSR II flow cytometer (BD Biosciences, San Jose, CA). Compensation and data analysis were performed using FlowJo software (TreeStar, Ashland, OR). After gating out cell aggregates, debris and dead cells, immune cells were identified using the pan-hematopoietic marker CD45. Specific cell types were identified as follows: alveolar macrophages (highly autofluorescent and CD11c^hi^CD11b^int^), interstitial macrophages (CD11b^+^CD11c^+^MHC II^+/−^), monocytes (CD11b^+^CD11c^−^Ly6C^+^MHC II^+/−^), neutrophils (CD11b^+^Ly6G^+^), B cells (CD19^+^), CD4 T cells (CD4^+^), CD8 T cells (CD8^+^), NK cells (NK1.1^+^), CD103^+^ dendritic cells (CD11c^+^CD11b^int^MHC II^+^CD103^+^), CD103^−^ dendritic cells (CD11c^+^CD11b^+^MHC II^+^CD103^−^). Data presented as percent of cells in CD45^+^ gate. Expression of the activation markers presented as median fluorescence intensity (MFI).

### Lung histopathology

We cut the inferior vena cava and perfused the right ventricle *in situ* with >1 ml of sterile PBS and then sutured a 20-gauge angiocath into the trachea via a tracheostomy. We then removed the lungs *en bloc* and inflated them to exactly 15 cm of H_2_O with 4% paraformaldehyde. We examined 5 µm sections from paraffin-embedded lungs stained with hematoxylin-eosin using light microscopy.

### Preparation of lung homogenates and viral plaque assay

We cut the inferior vena cava and perfused the right ventricle *in situ* with >1 ml of sterile PBS. We then removed the lungs, which were kept on ice prior to and during homogenization (Tissue Tearor, 30 s) in a flow cytometry tube with 1 ml of PBS. An additional 2 mL of PBS was added to the resulting homogenate and subjected to Dounce homogenization (20 strokes). The cell homogenate was centrifuged (4°C, 2000 rpm for 10 minutes). We grew MDCK cells in 6-well plates to 100% confluency. The MDCK cells were then treated with serial 10-fold dilutions of the supernatant in DMEM and 1% bovine serum albumin (BSA) and incubated for 1 hour (37°C). The inoculums were aspirated, washed with PBS, and 3 ml of replacement media [2.4% Avicel (FMC BioPolymer, Philadelphia, PA), 2× DMEM, and 1.5 µg of N-acetyl trypsin] was added to each well. The plates were subsequently incubated for 3 days. We then removed the overlay and visualized viral plaques using naphthalene black dye solution (0.1% naphthalene black, 6% glacial acetic acid, 1.36% anhydrous sodium acetate) [28].

### Statistics

We explored differences between groups using analysis of variance (ANOVA). When the ANOVA indicated a significant difference, we explored individual differences using *t* tests with a Dunnett or Tukey's correction for multiple comparisons as indicated. We performed all analyses using GraphPad Prism version 6.0 for Windows (GraphPad Software, San Diego CA, USA). Data are shown as means ± SE. A *p* value<.05 was considered statistically significant for all tests. All experiments were repeated three times.

## Results

### Obese mice with global deficiency of leptin receptor have worsened survival following influenza A infection

**Figure 1:**
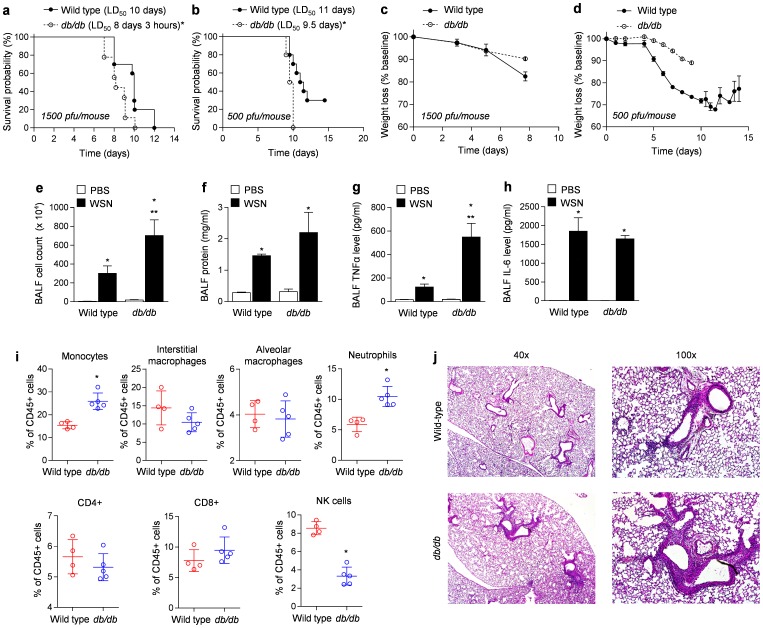
Effect of leptin receptor function on the survival, the degree of lung injury and inflammation during influenza A infection. *db/db* and C57BL/6 mice were inoculated with influenza A virus (A/WSN/33 [H1N1]) **(a)** 1500 pfu/mouse or **(b)** 500 pfu/mouse and mortality was subsequently assessed on a daily basis. Percentage of weight loss was also followed daily for the mice infected with **(c)** 1500 pfu/mouse or **(d)** 500 pfu/mouse. Four days after infection with influenza A virus 1500 pfu/mouse, *db/db* and C57BL/6 mice underwent a bronchoalveolar lavage for **(e**) assessment of cell count, **(f)** total protein, **(g,h)** inflammatory cytokines, **(i)** flow cytometry, and **(j)** the assessment of lung pathology (Hematoxylin and Eosin staining). * p<0.05 compared to PBS control. ** p<0.05 *db/db* compared to wild-type mice.

We intubated and infected *db/db* and wild-type mice with A/WSN/33 [H1N1] influenza A virus (500 and 1500 pfu/mouse) and followed them for up to 15 days after infection and recorded mortality. Compared to wild-type mice, mortality was significantly worse in the *db/db* mice infected with 1500 pfu/mouse (Figure 1a; LD_50_ 10 days versus 8 days and 3 hours in the wild-type and *db/db* mice, respectively, p<0.005) and 500 pfu/mouse (Figure 1b; LD_50_ 11 versus 9.5 days in the wild-type and *db/db* mice, respectively, p<0.05) of influenza A virus. The significant mortality observed in the *db/db* mice was not the result of weight loss as the wild-type mice lost more weight than the *db/db* mice (Figure 1c,d). Four days after inoculation with A/WSN/33 [H1N1] influenza A virus (1500 pfu/mouse), we observed an increase in BAL fluid cellularity (Figure 1e). The immune populations in the lungs of the wild-type and db/db mice were similar except there were a larger number of monocytes, and neutrophils and fewer natural killer (NK) cells in the BAL fluid of the *db/db* compared with wild-type mice (Figure 1e, i). TNF-α was also increased in the *db/db* mice compared with the wild-type mice but there was no significant difference in the levels of BAL fluid protein, IL-6, or the severity of lung injury evident upon careful examination of hematoxylin and eosin-stained lung sections (Figure 1f–h, j).

### Mice deficient in the leptin receptor have impaired viral clearance and diminished lung interferon-α levels after influenza A infection

**Figure 2:**
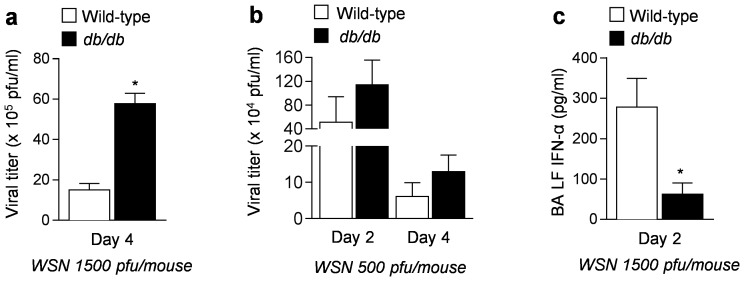
Effect of leptin receptor function on viral replication. Plaque forming units (pfu) were counted in MDCK cells treated with lung homogenates from *db/db* and C57BL/6 mice infected with influenza A virus **(a)** 1500 pfu/mouse on Day 4 and **(b)** 500 pfu/mouse on Day 2 and Day 4. We also measured **(c)** interferon-α (IFN- α) levels in BALF in wild-type and *db/db* mice 2 days after infection with influenza A virus (1500 pfu/mouse).

Four days after infection with influenza A virus (1500 and 500 pfu/mouse), we generated homogenates from the lungs of *db/db* and wild-type mice and measured the number of live viral particles using plaque assays. Elevated numbers of live viral particles were present in *db/db* compared with wild-type mice at both doses (p<0.05 for comparison between *db/db* and wild-type mice at Day 4 after infection with 1500 pfu/mouse) (Figure 2a,b). Compared to wild-type mice, *db/db* mice had lower levels of interferon-α (IFN-α) in their lungs 2 days after infection with influenza A virus (1500 pfu/mouse) (Figure 2c) suggesting that diminished interferon production may be responsible for the impaired viral clearance in *db/db* mice.

### The loss of leptin receptor signaling in the lung epithelium and macrophages is not associated with worsened lung injury or mortality following influenza A infection

**Figure 3:**
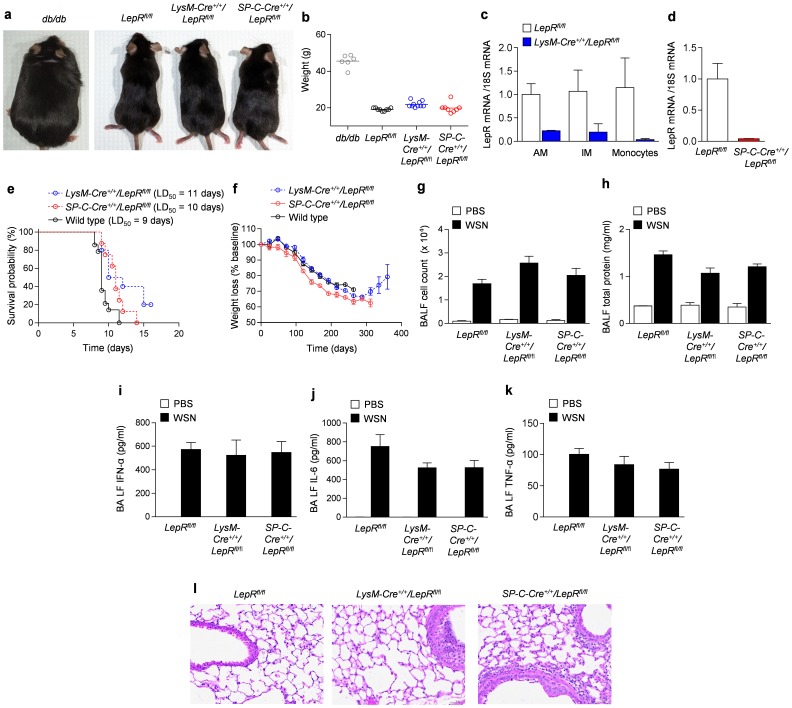
Effect of the leptin receptor function specifically within lung epithelium and within macrophages and neutrophils on survival, lung injury, and inflammation during influenza A infection. **(a)** Representative images and **(b)** weights of *db/db*, *LepR^fl/fl^*, *SP-C-Cre^+/+^/LepR^fl/fl^*, and *LysM-Cre^+/+^/LepR^fl^* ^/fl^ mice are shown. Quantitative real-time PCR reveals appropriate knockout of the leptin receptor **(c)** within the macrophages in *LysM-Cre^+/+^/LepR^fl^* ^/fl^ mice and **(d)** within the lung epithelium in *SP-C-Cre^+/+^/LepR^fl/fl^* mice. *LepR^fl/fl^*, *SP-C-Cre^+/+^/LepR^fl/fl^*, and *LysM-Cre^+/+^/LepR^fl^* ^/fl^ mice were inoculated with influenza A virus (500 pfu/mouse) with assessment of **(e)** mortality and **(f)** daily weight. Four days after infection with influenza A virus (500 pfu/mouse), *LepR^fl/fl^*, *SP-C-Cre^+/+^/LepR^fl/fl^*, and *LysM-Cre^+/+^/LepR^fl^* ^/fl^ mice underwent bronchoalveolar lavage for levels of **(g)** cell count, **(h)** total protein, **(i–k)** inflammatory cytokines and **(l)** lung pathology (Hematoxylin and Eosin staining).

We generated *SP-C-Cre^+/+^/LepR^fl/fl^* (mice with leptin receptor deficiency specifically within lung epithelium) and *LysM-Cre^+/+^/LepR^fl^* ^/fl^ mice (mice with leptin receptor deficiency specifically within macrophages and dendritic cells). In contrast to *db/db* mice, *SP-C-Cre^+/+^/LepR^fl/fl^* and *LysM-Cre^+/+^/LepR^fl^* ^/fl^ mice are non-obese at 8–12 weeks of age (Figure 3a and b). Quantitative real-time PCR revealed knockout of the leptin receptor within the macrophages in *LysM-Cre^+/+^/LepR^fl^* ^/fl^ mice and within the lung epithelium in *SP-C-Cre^+/+^/LepR^fl/fl^* mice (Figure 3c and 3d). We infected *LepR^fl/fl^*, *SP-C-Cre^+/+^/LepR^fl/fl^*, and *LysM-Cre^+/+^/LepR^fl^* ^/fl^ mice with A/WSN/33 [H1N1] influenza A virus (500 pfu/mouse). In contrast to *db/db* mice, both the *SP-C-Cre^+/+^/LepR^fl/fl^* and *LysM-Cre^+/+^/LepR^fl^* ^/fl^ mice survived longer than their wild-type controls (*LepR^fl/fl^*) following influenza A infection (Figure 3e; LD_50_ 9, 10 and 11 days for *LepR^fl/fl^*, *SP-C-Cre^+/+^/LepR^fl/fl^* and *LysM-Cre^+/+^/LepR^fl^* ^/fl^, respectively). However, there were no other detectable differences in the severity of lung injury in the three strains of mice as assessed by weight loss (Figure 3f), the number of leukocytes (Figure 3g) and protein (Figure 3h) in the BAL fluid, levels of the pro-inflammatory cytokines interferon-α, IL-6 or TNF- α (Figure 3i–k), or histologic examination of hematoxylin and eosin stained lung sections (Figure 3l).

### Mice with leptin receptor deficiency specifically within lung epithelium or within macrophages have improved viral clearance after influenza A infection

**Figure 4:**
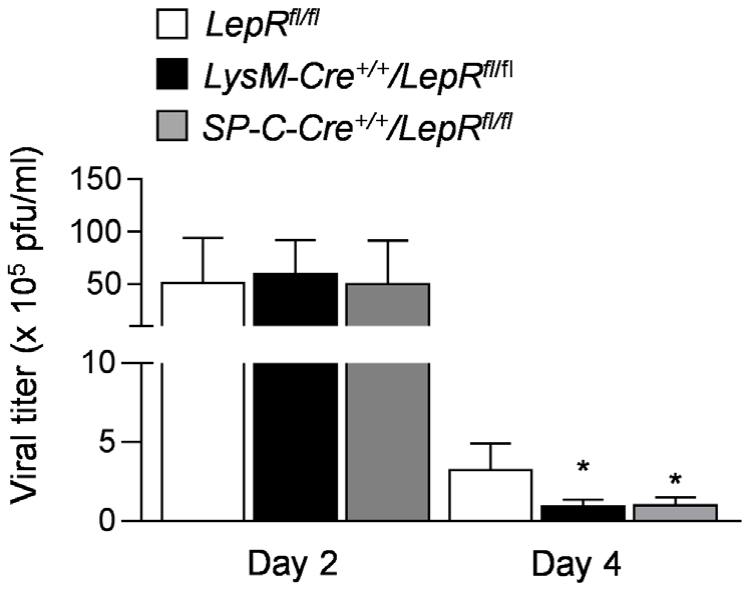
Effect of leptin receptor function specifically within lung epithelium and within macrophages and neutrophils on viral replication. Plaque forming units (pfu) were counted in MDCK cells treated with lung homogenates from *LepR^fl/fl^*, *SP-C-Cre^+/+^/LepR^fl/fl^*, and *LysM-Cre^+/+^/LepR^fl^* ^/fl^ mice infected with Influenza A virus 500 pfu/mouse on Day 2 and Day 4. * p<0.05 *LysM-Cre^+/+^/LepR^fl^* ^/fl^ and *SP-C-Cre^+/+^/LepR^fl/fl^* mice compared to *LepR^fl/fl^* mice.

We collected lung homogenates from *LepR^fl/fl^* (control), *SP-C-Cre^+/+^/LepR^fl/fl^*, and *LysM-Cre^+/+^/LepR^fl^* ^/fl^ mice 2 and 4 days after intratracheal administration of influenza A virus (500 pfu/mouse). Similar number of live viral particles were found in the lungs of all three strains of mice 2 days after infection with influenza A virus, however, the number of viral particles was significantly reduced in *SP-C-Cre^+/+^/LepR^fl/fl^*, and *LysM-Cre^+/+^/LepR^fl^* ^/fl^ mice compared to control mice on day 4 after infection (Figure 4).

## Discussion

In several large cohorts of patients hospitalized with H1N1 influenza infection during the pandemic that began in 2009, obesity was identified as a risk factor for adverse clinical outcomes [29]–[31]. Obesity and the associated metabolic syndrome are complex phenotypes characterized by diabetes, hyperglycemia, insulin resistance, fatty infiltration of the liver and acquired leptin resistance, all of which might influence the course of viral infection through overlapping or independent mechanisms. Here, we report that mice globally deficient in the leptin receptor, which exhibit many features of the metabolic syndrome, had impaired viral clearance and reduced survival following influenza A infection. To determine whether these changes might be attributed to reduced leptin signaling in the lung, we generated normal weight mice deficient in the leptin receptor in the lung epithelium or in lung alveolar type II cells, and macrophage/monocytes. Surprisingly, these mice showed a less severe lung injury phenotype and improved viral clearance following influenza A infection. These results suggest that the worsened outcomes we observed in the obese global leptin receptor knockout mice are independent of leptin signaling in these cellular compartments of the lung. Instead, leptin signaling in these cells impaired the clearance of the influenza A virus from the lung.

We and others have reported that mice globally deficient in leptin signaling are protected against oxidant-induced lung injury generated by exposure to hyperoxia and against the development of lung injury and fibrosis following the intratracheal administration of bleomycin [26], [32]–[34]. In these sterile models of lung injury, we suggested that leptin acted as a Type I cytokine in the lung to activate the innate immune system, thereby contributing to the development of lung injury and fibrosis. Here we report that these same animals have impaired viral clearance following infection with the influenza A virus. These results are consistent with those of Mancuso et al. who reported that leptin-deficient mice have impaired bacterial clearance and worsened lung injury in a murine model of *K. pneumonia* [35]. Similarly, Zhang et al. has recently reported that diet-induced obese mice had significantly higher initial pulmonary viral titer and mortality after challenge with A(H1N1)pdm09, compared with age-matched lean mice [36].

We reasoned that the resistance of the *db/db* mice to sterile lung injury and fibrosis and their sensitization to viral pneumonia might be attributable to loss of the Type I cytokine function of leptin in the lung. In support of this hypothesis, mice deficient in leptin signaling have been found to have defects in both cell-mediated and humoral immunity [37]–[39]. Alternatively, obesity itself or its metabolic consequences might explain the phenotype of these animals independent of leptin signaling [40]. To address this question, we took advantage of work by Friedman and colleagues, who created mice harboring floxed alleles in the genes encoding the leptin receptor and used them to show that leptin receptor deficiency in the brain is required and sufficient for the development of obesity and the related phenotypes associated with the metabolic syndrome in *db/db* mice [19]. We crossed these *LepR^fl/fl^* mice with mice expressing Cre recombinase driven by the *SPC* or *LysM* promoter to create mice lacking leptin receptors in cells in the lung important for the response to influenza A infection. Because leptin signaling in the brains of these animals was intact, they did not develop obesity or the associated metabolic syndrome. SPC is expressed early in the development of the lung epithelium, therefore, its developmental expression results in Cre mediated recombination in the entire airway and lung epithelium [41], [42]. The LysM-Cre animals express Cre recombinase in granulocyte and monocyte precursors in the bone marrow, which results in Cre mediated recombination in monocytes and macrophages [43]. In addition, the LysM promoter is expressed in mature alveolar Type II cells [44]. Surprisingly, we found that the loss of leptin signaling in either the lung epithelium or in the macrophages and alveolar type II cells was associated with improved viral clearance and a small reduction in the severity of the influenza A pneumonia. These results suggest that impaired leptin signaling in these cells in the lung cannot account for the impaired viral clearance and reduced survival we found in the global knockout mice. Instead, these abnormalities may be attributable to leptin signaling in other cellular compartments such as NK cells or T cells, obesity and/or other features of the associated metabolic syndrome. Our findings are consistent with those from other groups of investigators who have reported that mice with diet induced obesity have worsened lung injury and impaired viral clearance following influenza A infection [36], [45].

The influenza A virus preferentially infects alveolar type II cells, which express cell surface glycoconjugates containing terminal sialic acid residues that function as receptors for the hemagglutinin antigens of influenza A, promoting infection of the cells [46]. The virus then propagates in the alveolar type II cells and is released upon cell lysis. Because both of our tissue specific knockouts had very similar phenotypes in the influenza A pneumonia model and both promoters target alveolar type II cells, it is tempting to speculate that leptin signaling contributes to viral replication in these cells. In this regard, leptin, which is increased in the lungs of influenza A infected mice, can activate the JAK/STAT pathways, which might alter the expression of genes involved in the innate immune response to the virus or in the susceptibility of the type II cell to necrotic or apoptotic programmed cell death [47], [48]. While our targeted knockout strategies are not sufficiently specific to exclude a more complex mechanism or set of mechanisms to explain the observed phenotypes, our results suggest that studies using specific promoters that target adult alveolar type II cells can be performed without the obesity associated with global leptin deficiency.

The number and activity of macrophages are increased in the lungs of influenza A infected mice and play a major role in immune response against influenza A virus [49]–[51]. Macrophages have been reported to be the major source of interferon α/β, which plays a key role in viral clearance, and are important sources of IL-1β, IL-6 and TNF-α, which are responsible for the acute phase response to the infection [51]–[53]. Leptin receptors are expressed on macrophages where they may modulate the release of pro-inflammatory cytokines [54]. However, we were unable to detect significant differences in the lung levels of these cytokines between influenza A infected *LysM-Cre^+/+^/LepR^fl^* ^/fl^ mice and their controls. We found that viral clearance and lung injury severity were modestly improved and the number of inflammatory cells was similar in mice deficient in leptin signaling in macrophages and mature alveolar type II cells compared with controls. These findings were similar to those observed in mice deficient in leptin signaling in the lung epithelium, suggesting that leptin signaling in macrophages plays little role in the response to influenza A infection in mice.

Our study raises several questions about the mechanisms that underlie the increased sensitivity to influenza A infection in obese mice globally deficient in leptin signaling and perhaps in obese humans. It may be that leptin signaling in other cells important for the innate immune response to the virus might impair viral clearance. For example, leptin has been reported to play an important role in the activation of regulatory T cells during immunization and we observed reduced numbers of NK cells in *db/db* mice following infection with the influenza A virus [39]. These findings are consistent with those of other groups who have observed reduced NK cell recruitment and NK-mediated cytotoxicity in *db/db* mice [55], [56]. Alternatively, obesity itself or its effects on other tissues result in impaired viral clearance [40]. These questions can be addressed in further studies of mice with diet-induced obesity or by using the tissue specific knockout strategy we describe here to target other cellular components of the innate and adaptive immune system.

In summary, globally leptin receptor deficiency in obese mice demonstrated impaired viral clearance and reduced survival during influenza A pneumonia, mirroring clinical observations reported in obese humans. In contrast, non-obese, tissue specific knockout mice lacking leptin receptors in the lung epithelium and macrophages have improved viral clearance and slightly reduced lung injury following influenza A infection. We conclude that the loss of leptin signaling in these cells within the lung does not explain the reduced viral clearance and worsened mortality observed in the global knockout mice. Instead, leptin signaling in other cells within or outside the lung including T cells and NK cells or obesity and its associated metabolic consequences may be important for viral clearance following influenza A infection.
